# A practical starting guide to tear fluid biobanking

**DOI:** 10.1016/j.aopr.2026.04.001

**Published:** 2026-04-27

**Authors:** Jente Schmeetz, Suzanne Hagan, Nienke van de Sande, Nikolay Boychev, Karima Kessal, Tear Research Network Biobanking Taskforce, Alejandra de-la-Torre, William Rojas-Carabali, Erika Ponzini, Juanita Cardona-López, Imre Lengyel, Sarah B. Sunshine, Carlos Cifuentes-González, Françoise Brignole-Baudouin, Swaminathan Sethu, Marlies Gijs

**Affiliations:** i Neuroscience Research Group (NEUROS), Neurovitae Center for Neuroscience, Institute of Translational Medicine (IMT), School of Medicine and Health Sciences, Universidad del Rosario, Bogotá, Colombia; j Colombian Visual Science and Translational Eye Research Institute (CERI), Bogotá, Colombia; k Department of Materials Science, University of Milano-Bicocca, Via R. Cozzi 55, Milano, 20125, Italy; l COMiB Research Centre in Optics and Optometry, Via R. Cozzi 55, Milano, 20125, Italy; m The Wellcome-Wolfson Institute for Experimental Medicine, School of Medicine, Dentistry and Biomedical Science, Queen's University Belfast, 97 Lisburn Road, Belfast, BT9 7BL, UK; n Department of Ophthalmology and Visual Sciences, University of Maryland School of Medicine, Baltimore, MD, USA; o University of Maryland Greenebaum Comprehensive Cancer Center, University of Maryland School of Medicine, Baltimore, MD, USA; p Ocular Immunology Department, Ophthalmology Research Center, SIGMA Clinic, Cali, Colombia; q Paris cité university, Faculty of Pharmacy, Paris, F-75006, France; r GROW Research Laboratory, Narayana Net-ralaya Foundation, Bangalore, India; s Sorbonne Université, INSERM, CNRS, Institut de la Vision, Paris, France; t Hôpital National de la Vision des 15-20, INSERM-DGOS CIC 1423, IHU FOReSIGHT, Paris, France; a University Eye Clinic, Mental Health and Neuroscience Research Institute, Maastricht University, Maastricht, the Netherlands; b Department of Vision Sciences, School of Health & Life Sciences, Glasgow Caledonian University, Scotland, G4 0BA, UK; c Department of Ophthalmology, Massachusetts Eye and Ear, Boston, MA, USA; d Department of Ophthalmology, Harvard Medical School, Schepens Eye Research Institute at Massachusetts Eye and Ear, Boston, MA, USA; e Department of Clinical Education and Clinical Sciences, New England College of Optometry, Boston, MA, USA; f Sorbonne Université, INSERM, CNRS, Institut de la Vision, Paris, France; g Hôpital National de la Vision des 15-20, Service d'ophtalmologie 3, IHU FOReSIGHT, Paris, France; h Hôpital National de la Vision des 15-20, INSERM-DGOS CIC 1423, IHU FOReSIGHT, Paris, France

**Keywords:** Tear fluid, Storage, Biobank, Guidelines

## Abstract

**Background:**

Tear fluid is increasingly recognized as a minimally invasive source of biomarkers in various diseases with broad potential for diagnostics and disease monitoring. Biobanking tear fluid samples provides valuable access to high-quality biological samples while reducing the need for new collection efforts. Ensuring appropriate collection and storage conditions is critical for maintaining sample integrity. However, standardized guidelines and operating protocols for tear fluid biobanking are still lacking.

**Main text:**

This review synthesizes existing literature on tear fluid biobanking, complemented by additional sources such as reports and online guidelines on biobanking standards and recommendations. Together, these insights provide a practical guide for researchers and institutions aiming to establish a tear fluid biobank or integrate tear fluid samples into existing biobanking infrastructures. This guide outlines key aspects including (i) governance, regulatory, and ethical considerations, (ii) biosafety, infrastructure, and logistics, (iii) collection methods and materials, and (iv) data harmonization through the developed and proposed Tear Fluid-Common Data Elements, which emphasize on the collection, storage, processing, and analysis of biobanked samples.

**Conclusions:**

By providing clear and practical guidance, this review supports the standardization of tear fluid biobanking, thereby enhancing the value of these samples for future research and clinical applications.

## Highlights

• Biobanking tear fluid offers access to high-quality samples with fewer new collections.
• Practical guidance is provided for setting up a dedicated tear fluid biobank.
• Key considerations span governance, ethics, biosafety, infrastructure, and logistics.
• Tear Fluid-CDEs are presented to harmonize collection, storage, and analysis workflows.

## 1 Background

Biomedical research and medicine have evolved to address healthcare needs by using more personalized approaches.^1^ In this context, human biofluids have become increasingly important in identifying novel biomarkers for diagnostics and targeted therapeutics.^2^^,^^3^ In biobanks, also referred to as biorepositories, a great variety of biofluids and tissue samples are stored, including, among others, serum, plasma, urine, cerebrospinal fluid (CSF), skin, and brain tissues.^4^^,^^5^ In recent years, tear fluid has emerged as a biofluid of growing interest for research and biobanking.^6^, ^7^, ^8^ Tear fluid is easily accessible, and its collection is minimally invasive compared to other biofluids, such as blood and CSF.^9^^,^^10^ Analyzing tear fluid samples is becoming increasingly feasible because of the rapid advances in detection techniques.^11^ For example, in the 1990s, proteins could be detected using Western Blot or the first enzyme-linked immunosorbent assays (ELISA) that had detection limits in the microgram range. By the 2000s, sensitivity improved to the picogram level, and by the 2020s, techniques have reached the femtogram and attogram range and can achieve single-molecule analysis.^12^ These technological advancements enable the detection of low-abundance biomarkers that were previously undetectable.^11^ Beyond proteins, tear fluid samples allow for the analysis of various other molecular constituents, including RNA, microRNAs, DNA, lipids, metabolites and extracellular vesicles.^11^ In addition, more sophisticated technologies allow for the analysis of small sample volumes, which was previously a major limitation, thereby expanding research possibilities.^11^ As a result, tear fluid is a promising biofluid with great potential for diagnostic and therapeutic applications in human pathologies.^11^ Therefore, storing tear fluid samples is of significant interest for ongoing and future research.

Although interest in tear fluid biomarker research is growing, to our knowledge, only a limited number of biobanks worldwide currently include tear fluid samples or are specifically designed for the storage of this biofluid. The limited representation of tear fluid samples in biobanks may stem from various hurdles, including the lack of standardized protocols for collection and storage, the specialized equipment and technical expertise required, and varying awareness and engagement among clinicians and researchers.^11^ These challenges have prompted researchers to establish local, study-specific tear fluid storage practices. Consequently, there is a clear need for practical guidelines to also standardize local biobanking approaches.

To support the integration of tear fluid samples into existing and new (local) biobanks, this review provides a practical starting guide for researchers or clinicians seeking to initiate tear fluid storage within an established biobank or to establish a new biobank. To support this effort, general biobanking principles, including relevant rules, regulations, and ethical considerations, as well as considerations specific to the collection, handling, and storage of tear fluid samples are comprehensively discussed.

## 2 Biobank types

**Fig. 1:**
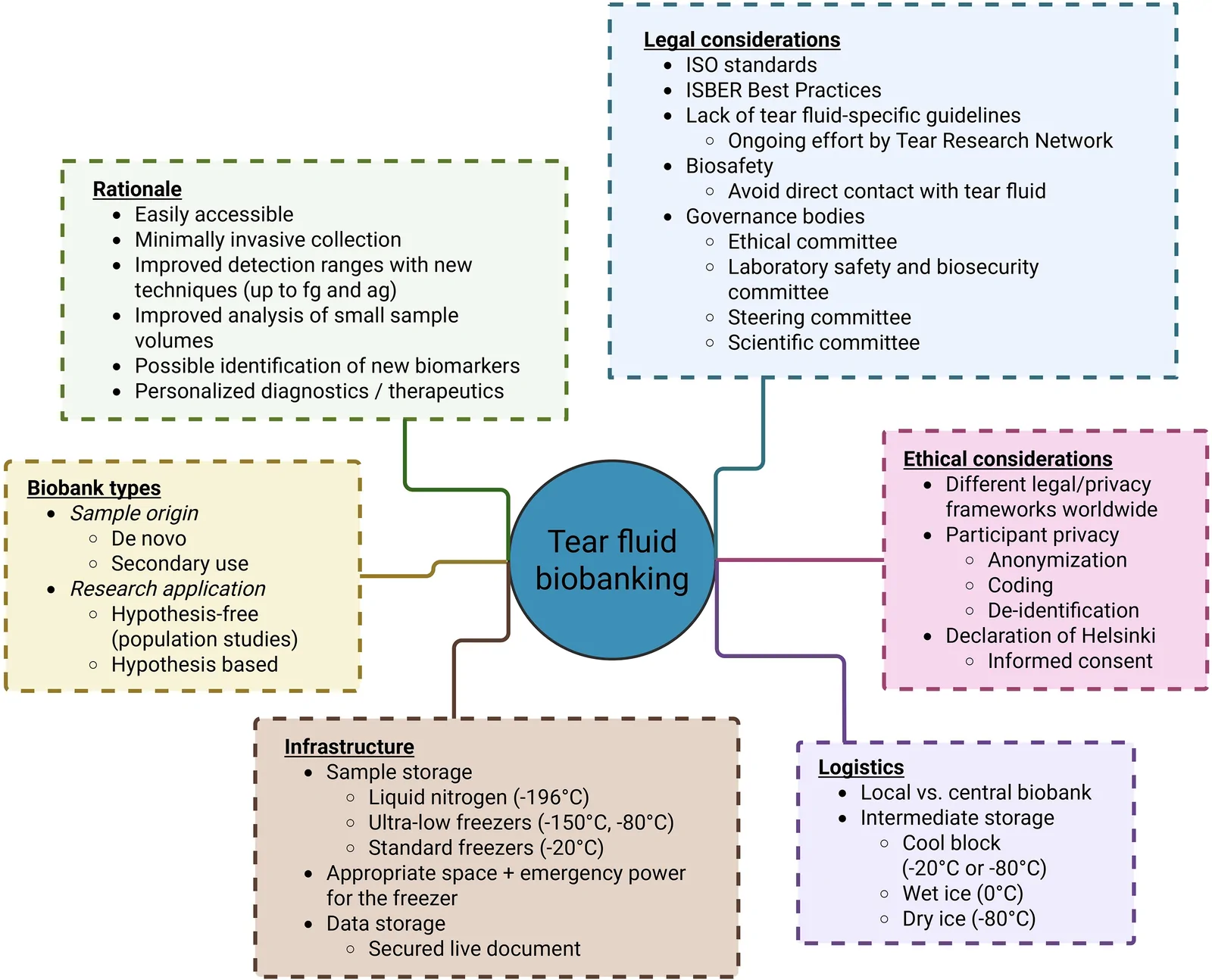
Core concepts of tear fluid biobanking, including different types of biobanks, infrastructure, logistics, and legal and ethical considerations.

Biobanks are specialized facilities that collect and store biofluids and tissues for clinical and research purposes.^13^ By enabling access to a wide variety of biological samples, biobanking supports the generation of high-quality, reliable data.^13^ Depending on the sample origin, biobanks can be broadly classified into two main types: secondary-use biobanks and de novo biobanks^14^ (Fig. 1). A secondary-use biobank stores biological samples collected initially for its original purpose, typically follow-up diagnostics, that are subsequently repurposed for research. In contrast, a de novo biobank contains samples that are prospectively collected with the explicit intention of supporting research. Another way to classify biobanks is based on the intended research application. Hypothesis-free or untargeted biobanking involves collecting and storing samples without a predefined research question, applying minimal inclusion and exclusion criteria.^15^ This allows for retrospective selection from a generally large database of samples for various research purposes. As an example, population studies focus on deep phenotyping of typically healthy individuals and generate extensive datasets that can be linked to stored samples.^15^ Large-scale population studies, such as the Maastricht Study,^16^ the Rotterdam Study,^17^ LifeLines,^18^ UK Deep Phenotyping Study,^19^ and the UK Biobank,^4^ exemplify this approach. Another research application involves hypothesis-based or targeted biobanks, which store samples collected from preselected cohorts based on strict inclusion and exclusion criteria.^15^ These studies aim to answer predefined research questions. Once all subjects are included, the samples are systematically analyzed, which often leads to sample depletion. Understanding these different biobanking strategies is crucial for establishing an effective biobank that aligns with the defined research goals and ensures its utility for future research.

## 3 Legal considerations

### 3.1 Governance

Compliance with quality guidelines is crucial for maintaining sample integrity, and to define its use, it is recommended for biobanks to establish clear governance structures to effectively manage operational costs, secure consistent funding, ensure long-term sustainability, support day-to-day practices, and supervise the granting of samples and associated data (Fig. 1). The governance framework may comprise three bodies: (i) a steering committee to oversee the management of the biobank and define its scientific and strategic development priorities, (ii) a scientific (or strategic) committee responsible for the development of new research projects and evaluating requests for access to samples, and (iii) laboratory safety and biosecurity committee to ensure quality control and compliance. The specific committees involved may vary depending on the biobank and the specific institutional requirements.

### 3.2 Regulatory frameworks

Collecting and storing tear fluid samples as part of a biobank is subject to several rules and regulations designed to ensure secure storage and responsible management of biological materials and associated data. Several International Organization for Standardization (ISO) standards have been developed to guide the biobanking of biological resources^20^^,^^21^ (Table 1). Compliance with these ISO standards serves as a framework for quality management, thereby ensuring that biobanks operate according to best practices.^20^ In addition, the International Society for Biological and Environmental Repositories (ISBER) has established a best practices guideline for managing and operating biobanks (ISBER Best Practices: Recommendations for Repositories, Fifth Edition).^22^ Complementing these recommendations, Carranza et al. recently published a review on biological sample storage in biomedical research, providing practical guidance for transforming general sample storage into a formal biobank and outlining the steps required for this transition.^5^

**Table 1:** ISO standards for biobanking biological resources.^21^

ISO standard	Scope
ISO 20387:2018	General requirements for biobanking, including the establishment, implementation, maintenance, and continuous improvement of quality management systems within biobanks.
ISO/TR 22758:2020	Implementation guide for ISO 20387.
ISO 21899:2020	Requirements and guidance for the validation and verification of processing methods used in biobanks.

While existing ISO standards and best practice guidelines provide a framework for general biobanking, there are currently no specific standards available for biobanking tear fluid samples. Biofluid-specific Standard Operating Procedures are essential to ensure the quality and integrity of samples throughout the collection and storage. Although established protocols exist for handling other biofluids, such as blood,^23^ no equivalent protocols are currently available for tear fluid. This highlights the need for best practice guidelines for the handling and storage of tear fluid samples, which is an ongoing effort by the Tear Research Network.^24^

### 3.3 Sample and data sharing

Biospecimens and associated data are generally not permitted for sale or commercial exchange, unless explicitly allowed by biobank-specific policies or in the case of commercial biobanks. When samples or data are shared with external parties, appropriate agreements, such as Material Transfer Agreements for the exchange of biological samples and Data Sharing Agreements for sharing data in accordance with data protection laws, must be in place to ensure ethical and legal use. Finally, the presence of DNA in tear fluid samples may trigger stricter regulatory requirements. In such cases, national and/or institutional guidelines specifically related to the handling, storage, and use of genetic material must be followed to ensure compliance with national legal regulations.

### 3.4 Biosafety

Adherence to appropriate biosafety guidelines, designed to protect personnel from potential exposure to infectious agents, is another critical consideration in biobanking biofluids. However, research on the biosafety of tear fluid remains limited. Published reports suggest that tear fluid does not support the transmission of HIV,^25^^,^^26^ and that the risk of SARS-CoV-2 transmission via tear fluid appears to be low.^27^^,^^28^ However, adenoviruses can be transmitted through tear fluid, indicating a possible, although low, risk of exposure.^29^ For safety reasons, standard biosafety guidelines recommend using gloves, or alternatively, safe handling tools such as tweezers, to avoid direct contact when working with any human body fluid, including tear fluid.^30^ Biosafety precautions remain critical to responsible biobanking practice, ensuring staff safety and sample integrity (Fig. 1).

## 4 Ethical considerations

Responsible use of biological samples and associated data is a key ethical consideration in biobanking 31, 32, 33. Although biobanking has become increasingly integrated into healthcare research, governance standards regulating these practices vary widely between countries.^34^ Similarly, privacy considerations, such as data protection frameworks, differ worldwide. In Europe, biobanks must comply with the General Data Protection Regulation (GDPR).^35^ At the same time, in the United States, privacy in biobank-based research is governed by the Health Insurance Portability and Accountability Act (HIPAA) Privacy Rule.^36^

Participant privacy can be achieved through anonymization, coding, or data de-identification. Anonymized data must not contain direct or indirect identifiers and cannot be linked to individuals by anyone, including the researchers.^37^ Coded data retain a link to the participant through a unique code stored separately from personal identifiers.^37^ De-identified data minimize the risk of re-identification by removing the links to the participants.^37^ The choice of privacy protection approach depends on the biobank's purpose and relevant privacy regulations.

Ethical considerations regarding medical research involving human participants, samples, or data are guided by international frameworks such as the Declaration of Helsinki, which emphasizes informed consent and ethical review.^38^ Ethical review refers to the approval granted by an independent ethical committee for the biobank and its planned procedures, including the essential approval of the informed consent form. This form must contain information about sample collection, the biobank's purpose, types of samples collected, and potential current and future uses, including data and sample sharing and data protection measures. It should also outline any potential risks (e.g., minor discomfort during tear fluid collection), emphasize voluntary participation and withdrawal rights, and provide clear contact information to ensure transparency and ethical compliance. Informed consent must be obtained from participants prior to their participation in the collection of tear fluid for biobanking purposes.

Since ethical approval, data protection, biosafety standards, and governance differ between countries, it is essential to understand the legal and ethical frameworks of the country where the biobank will be established. Consultation with local regulatory authorities, institutional review boards, and ethics committees is strongly advised to ensure regulatory compliance.

## 5 Infrastructure

### 5.1 Tear fluid sample storage

Maintaining the integrity of tear fluid samples depends on adherence to the appropriate freezing and handling procedures. Storage options range from liquid nitrogen (−196 °C/−320.8 °F) and ultra-low freezers (−150 °C/−238 °F or −80 °C/−112 °F) to standard freezers (−20 °C/−4 °F), each offering different levels of preservation depending on the intended duration and type of analysis. No standardized guidelines have yet been established for optimal storage temperatures across different durations and types of analyses. However, ongoing efforts by the Tear Research Network aim to develop such guidelines. In the current absence of standardized protocols for tear fluid, it is recommended to follow general principles of biological sample preservation, including minimizing time between collection and storage, maintaining temperature-controlled conditions, and storing samples at low temperatures.

To prevent sample loss due to power failure, temperature monitoring systems that trigger alarms when the temperature deviates from the set range are required, along with emergency power sources such as backup generators.^22^ Furthermore, it is recommended to place the biobank freezer(s) in dedicated, well-ventilated storage areas to minimize the noise and heat generated, particularly those operating at ultra-low temperatures. Additionally, freezer selection should be guided by the available space and type of unit. Upright versus chest freezers offer different advantages with regard to accessibility and storage capacity. Freezers with easily accessible drawers and storage racks can help minimize the frequency of door openings, thereby reducing temperature fluctuations and preserving sample quality. Appropriate storage boxes are critical to fit the specific tube sizes used during the collection of tear fluid. Combined with assigned box positions that correspond to the database and freezer overview, these measures ensure a storage system that remains organized and maintainable. In this storage system, an advantage of tear fluid samples is the relatively small storage space required compared to other biological fluids. To ensure sample security, the freezer should be located in a locked room with restricted access for authorized personnel only. Lastly, the biobank freezer must be used exclusively for research samples, indicating that no food or unrelated items should be stored inside.

### 5.2 Tear fluid sample data storage

Accurate and secure tracking of collected samples is essential, typically achieved through the use of a screening and enrollment list, which is maintained as a live document or a master sheet. At a minimum, the screening and enrollment list may include the sample code, participant code, collection date, eye laterality, sample type and volume or migration length (for Schirmer's strips), and exact storage location (i.e. freezer and box number). In this document, all data is coded, with the key stored in a separate secure document that connects each code to the corresponding identifiers. Furthermore, it should be possible to link the sample to its storage location, the clinical and epidemiological data, and the analytical outputs. To ensure proper sample tracking, storage tubes can be provided with a linear barcode or quick response (QR) code containing a unique identifier. This sample labeling method is particularly relevant for hypothesis-free biobanking, and may be especially suited to centralized biobanks, as it requires specific equipment such as printers with associated software or barcode scanners for labeled tubes. All data should be managed using secure, password-protected software that complies with applicable data protection regulations (e.g., GDPR or HIPAA).

## 6 Logistics

**Fig. 2:**
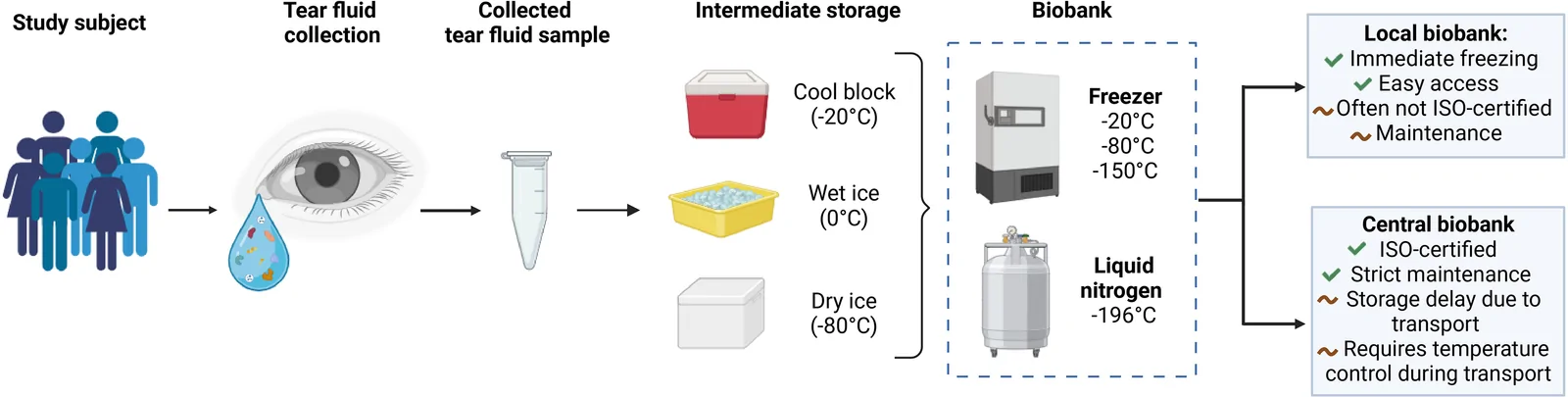
Schematic presentation of the tear fluid biobanking workflow. Tear fluid is collected from study participants and placed in a tube. Samples may be temporarily stored using intermediate cooling methods (cool block, wet ice, or dry ice) to maintain quality before transfer to the biobank. Within the biobank, samples are preserved in freezers or liquid nitrogen, either at a local or central facility.

In tear fluid biobanking, decisions about where and how to store samples are crucial to ensure quality and accessibility. Samples can either be stored in a local biobank (e.g. at the department in the hospital or university) or transported to a centralized biobank (Fig. 2). Each option offers its own set of advantages and disadvantages. For local storage, prompt freezing can be achieved by transporting the sample tubes immediately to a storage freezer, ideally within minutes of collection. Freezing shortly after sample collection helps maintain the stability of molecular components, such as cytokines and chemokines, and prevents microbial growth, thereby reducing the risk of sample degradation during transport.^39^ Furthermore, local storage provides researchers within the institution with ease of access. However, local freezers are often not ISO-certified, and long-term maintenance, including frequent defrosting, temperature monitoring, and backup power, may be less reliable.

Centralized biobanks typically operate under strict quality control standards, often with ISO certification, ensuring standardized sample handling, tracking, and optimal long-term storage conditions.^39^ However, transporting samples to a centralized facility can introduce delays, which may result in the gradual breakdown of molecules, particularly proteins, which can compromise the quality of the samples.^40^ Minimizing these delays between collection and storage is critical for maintaining sample quality and preserving sample integrity for future analyses. Therefore, the choice between local and centralized storage depends on the study design, transport considerations, logistical factors, and available resources.

If the storage freezer is not located near the collection site or tear fluid samples are collected during home visits, intermediate (temporary) freezing methods are necessary to preserve sample integrity before long-term storage in a freezer (Fig. 2). A cool block, such as a pre-cooled benchtop cooler, can maintain low temperatures and protect sensitive components in the sample. Wet ice (i.e., regular ice, frozen water) can also be used for temporary storage but requires frequent replenishment due to its rapid melting. Dry ice (solid CO_2_) provides a more stable freezing environment and melts less rapidly than wet ice, making it a preferable option when available. However, the limited availability of dry ice at collection sites and the release of CO_2_ in confined spaces can pose increased challenges to the collection of tear fluid. Any modification or breakdown in the cold chain must be recorded in the live master sheet.

Regardless of the chosen method, ensuring a continuous cooling chain by frequently renewing the cool block or ice throughout the day is essential, as these cooling solutions cannot be left unattended for extended periods. Further investigation, such as ongoing efforts by the Tear Research Network, are required to determine the optimal method for temporary storage between collection and storage in the freezer.

## 7 Tear fluid collection, processing and storage

### 7.1 Tear fluid collection methods and materials

**Fig. 3:**
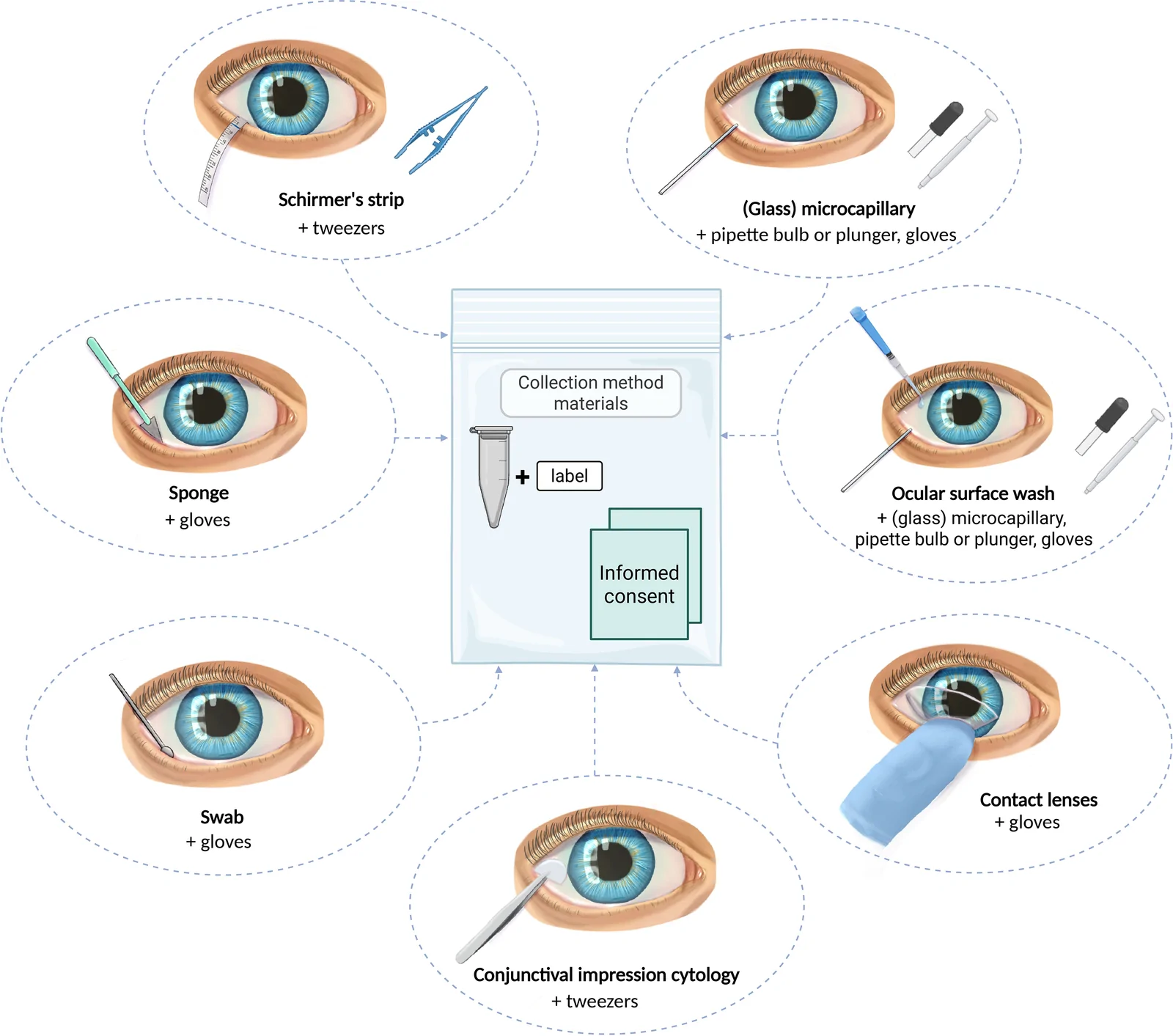
Materials required for tear fluid collection, listed by collection method. Pre-labelled tubes and informed consent are required for each collection method. Gloves are optional for all methods.

For the molecular analysis of tear fluid, there are multiple collection methods in use, ranging from Schirmer's strips and (glass) microcapillary tubes to sponges, swabs, ocular surface wash, conjunctival impression cytology, and contact lenses (Fig. 3).^11^ Based on the literature between 2020 and 2024, Schirmer's strips were the most commonly used collection method, accounting for approximately 40% of cases, followed by microcapillary tubes at 35%.^11^ Both methods have distinct advantages and limitations. Schirmer's strips are inexpensive and easy to use; however, they can cause ocular surface irritation upon removal, stimulate reflex tearing, and may collect conjunctival cells.^41^^,^^42^ Capillary tubes minimize contact with the ocular surface, thereby reducing conjunctival irritation and cell contamination.^43^ However, the capillary-collection process is relatively slow, requires well-trained, skilled hands, and does not apply to all participant groups, which can limit its clinical applicability.

The materials and handling protocols required for the removal of the collection method from the eye are specific to each method (Fig. 3). For example, for removing Schirmer's strips from the eye the use of sterile tweezers is recommended to minimize direct contact with the tear fluid sample and thereby ensure sample purity. The packaging of Schirmer's strips also affects its handling; individually packed strips reduce contamination risk, whereas paired strips must be carefully separated to avoid accidental contact. For (glass) microcapillary tubes, a plunger is required to efficiently expel the collected tear fluid into the storage tube. More generally, gloves use is recommended for all collection methods to maintain a clean environment, prevent sample contamination, and protect personnel from potential exposure. In the case of (glass) microcapillary tubes, swabs, and sponges, wearing gloves is particularly important, as it helps preserve sterility during the transfer of samples into storage tubes (Fig. 3). Similarly, when using contact lenses for tear fluid collection, careful removal while wearing gloves is essential to minimize the risk of sample loss or contamination during transfer (Fig. 3).

The choice of collection method may significantly impact the molecular composition and volume of the collected sample.^11^ In addition, the preferred method for tear fluid sampling may depend on the target patient group and the type of subsequent analysis.^11^ However, standardized recommendations regarding optimal collection methods for specific analytical purposes (i.e., required molecular compositions) and specific patient populations remain lacking. This gap underscores the need for further research, including ongoing efforts by the Tear Research Network.^24^ Until standardization efforts have yielded definitive recommendations on the optimal collection method, and recognizing that specific patient populations, analytes, or outcomes of interest may require different collection methods, biobanks are advised to maintain the capacity to store tear fluid samples collected using multiple methods.

In the context of targeted biobanking, maintaining consistency throughout sample collection is crucial once a collection method has been selected. Switching methods may introduce variability, which is difficult to account for in subsequent sample analyses. In hypothesis-free biobanking, variation in collection methods may occur. However, such variations should be carefully documented, as biomarker levels derived from different collection methods may not be directly comparable. Furthermore, tear fluid volumes may vary between manufacturers of the same collection method, as reported for Schirmer's strips.^44^^,^^45^ These observations underscore the importance of methodological consistency.

### 7.2 Tear fluid sample processing prior to storage

Following tear fluid collection, key data of each sample should be documented in an overview file. This includes details such as wetting/migration length on the Schirmer's strip (mm per x minutes) or collected tear fluid volume for e.g. (glass) microcapillary collection, types of tear fluid collected (i.e. basal tear fluid, reflex tear fluid, emotional tears), eye laterality, time of collection (e.g., morning or afternoon), and duration of the collection process. Furthermore, it is important to record whether the sample came into contact with the skin or if the collection method shows any visible traces of makeup. Currently, it is unclear whether these factors interfere with the measurement of biomarkers in tear fluid. Therefore, we recommend biobanking these valuable samples, while carefully documenting such occurrences to allow for potential data correction. Accurate and consistent documentation supports data quality and reproducibility, and should align with proposed common data elements (CDEs), based on established tear fluid-specific reporting guidelines (TEAR-RG).^46^

Tear fluid samples are often biobanked without prior processing. For all collection methods, however, samples can generally be stored under two conditions: either dry, with a buffer added only during tear fluid extraction, or with the collection device placed directly into a buffer solution for storage, prior to tear fluid extraction. In the case of Schirmer's strips, the entire strip is typically stored without the addition of any buffer. Depending on the experimental plan, extracting tear fluid from the strip before storage could enable aliquoting, though this is generally considered unfeasible due to the small sample volumes retrieved and the need for a nearby laboratory. Furthermore, it would add complexity to the workflow. For samples collected using (glass) microcapillaries, tear fluid can be diluted in a buffer before being stored in the freezer. Due to the limited sample volume, aliquoting these samples is challenging. The small volume of tear fluid samples also often results in minimal residual material available for storage after initial analysis. However, recent advances in detection technologies now enable reliable analyses using only a few microliters of sample. This development creates opportunities for the biobanking of remaining tear fluid, and we strongly recommend doing so whenever possible.

### 7.3 Storage of collected tear fluid samples

Appropriate tube size selection is critical for sample storage to ensure the collected volume fits and the tubes can be securely sealed and labeled, thereby minimizing the risk of sample loss. For example, for Schirmer's strips, centrifuge tubes with a minimum volume of 1.5 mL are recommended to ensure the strip fits without needing to fold the strip excessively. Alternatively, tubes with an insert for small volumes are suggested for tear fluid collected using microcapillaries due to the small volume. For ultra-low temperature storage, such as in liquid nitrogen or at −150 °C, screw-cap tubes (e.g., 2.0 mL cryovials used for cell culture) are recommended to ensure secure long-term preservation. Low retention tubes are recommended for all storage conditions to minimize protein adherence to the tube walls and reduce sample loss. Tear fluid is typically collected from both eyes, but stored separately, requiring two tubes per participant per collection point. Consequently, each sample must be labeled with the correct eye designation (i.e., OD for the right eye (oculus dexter) and OS for the left eye (oculus sinister)). Furthermore, in the case of study-specific biobanking, each sample label should contain at least the study name or abbreviation, participant number (in case of coded samples), and visit or sample number if applicable. In the context of general, typically centralized, long-term biobanking, each sample should be labeled with a linear barcode or QR code containing a unique identifier to ensure proper tracking of samples. Cryogenic stickers are recommended for labeling stored samples, as they can withstand extremely low temperatures and prevent labels from detaching from the tubes, which could otherwise result in unlabeled samples. Furthermore, pre-printed labels are preferred over handwritten ones to ensure clarity and readability. Alternatively, tri-coded tubes may also be used, though they require appropriate equipment for barcode reading.

### 7.4 Example of a tear fluid collection procedure using Schirmer's strips

**Fig. 4:**
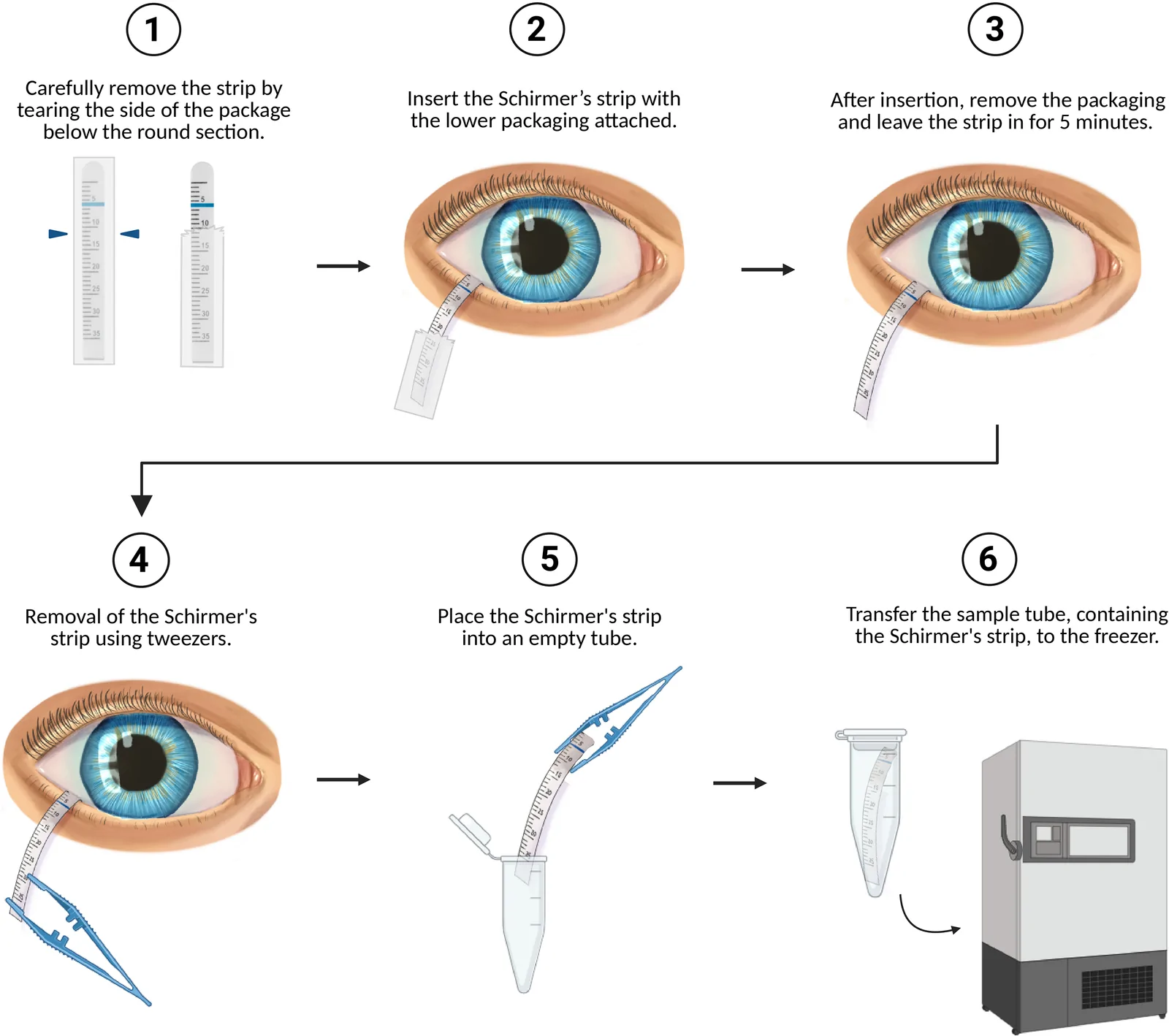
Tear fluid sampling protocol using Schirmer’s strips.

Schirmer's strips are widely used to assess tear fluid production, particularly in the diagnosis of dry eye. It can also serve as a method for collecting tear fluid. Here, an example of the procedure for collecting tear fluid using Schirmer's strips is provided. Before starting the tear fluid collection, gather the necessary materials, including a Schirmer strip, tweezers, a labeled storage tube, and a designated file for recording the migration length and the test duration. The Schirmer strip must be carefully removed from its sterile packaging to prevent contamination by direct contact with the strip. This can be accomplished by tearing the side of the package just below where the circular end of the strip is located (Fig. 4, step 1). Then, this small section of the packaging can be ripped off, exposing the circular end of the strip. This allows for the strip to be positioned in the eye while the lower part of the strip remains within its protective packaging. The Schirmer strip is typically placed into the lower conjunctival sac, approximately 2/3rds from the medial canthus of the participant's eye (Fig. 4, step 2). Once the strip is gently inserted, thereby avoiding reflex tearing, the remaining packaging should be removed from the strip. It is recommended for the participant to keep his/her eyes closed for the entire duration that the strip remains in the eye. Once 5 min have passed, or if the strip has become fully saturated before the 5-min cutoff (which should be recorded, and may potentially indicate reflex tearing), it should be carefully removed from the eye using sterile tweezers (Fig. 4, step 4). After placing the Schirmer's strip in the pre-labeled tube, it can be stored in the −80 °C freezer (Fig. 4, step 6). Due to the potential contribution of the strip to background signal during analysis, some researchers retain the entire strip to ensure consistency in strip length, and consequently background signal, across samples.^44^ Others, on the other hand, prefer to trim the non-wetted portion prior to storage to improve the precision of quantification. At present, there is no clear evidence favoring one approach over the other, indicating that further investigation is needed.

### 7.5 Example of a tear fluid collection procedure using (glass) microcapillary tubes

**Fig. 5:**
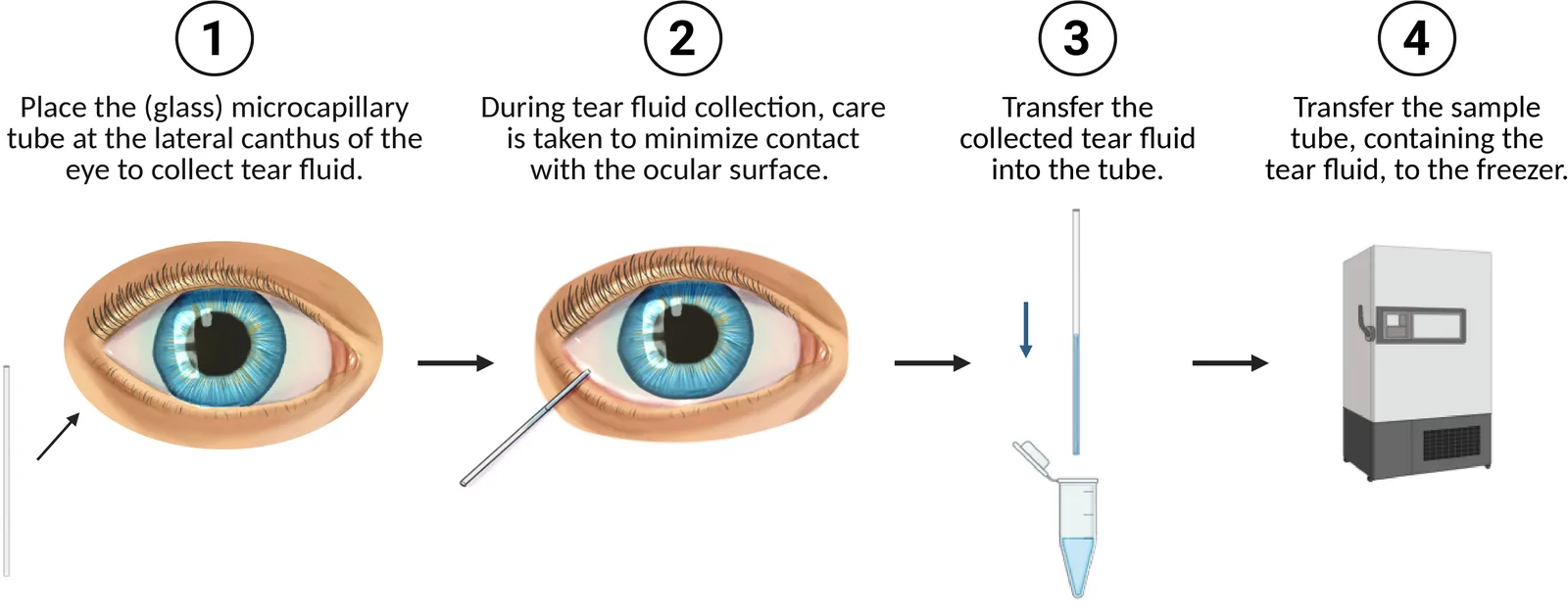
Tear fluid sampling protocol using (glass) microcapillary tubes.

Tear fluid collection using (glass) microcapillary tubes is another non-invasive method. The following outlines an example collection procedure. Before starting the tear fluid collection, the following components are required: a (glass) microcapillary, a pipetting bulb or plunger, and a pre-labeled, low-retention tube. To begin tear fluid collection, place the (glass) microcapillary at the lateral canthus of the subject's open eye while wearing gloves (Fig. 5, step 1). Tear fluid is gently collected by capillary action, while ensuring minimal contact with the ocular surface (Fig. 5, step 2). The collected tear fluid sample is then transferred into a pre-labeled tube by expelling the fluid from the capillary using a pipetting bulb or plunger (Fig. 5, step 3). The tear fluid samples are subsequently stored in the freezer at −80 °C (Fig. 5, step 4).^7^^,^^8^

## 8 Data science considerations and common data elements

The long-term scientific value of tear fluid biobanks depends not only on sample preservation but also on the rigor of data storage and analysis. From a data science perspective, several domains require attention to ensure that biobanked material can support sustained scientific relevance and reproducibility. Tear fluid biobanks are recommended to adopt structured standards like the CDEs,^47^ to harmonize clinical, demographic, and biospecimen data. Recently, the Tear Research Network published the TEAR-reporting guidelines (TEAR-RG), developed through an international Delphi consensus, which identified the critical variables that must be reported in tear fluid research.^46^ Building on these guidelines, a set of Tear Fluid-CDEs is proposed here to structure the minimum of data to be collected by tear fluid biobanks. These Tear Fluid CDEs are divided into two categories: (1) CDEs related to the collection and storage of samples (i.e., all steps prior to biobank storage) (Table 2), and (2) CDEs related to the processing and analysis of biobanked samples (Table 3). The latter may also be valuable to record in biobank data, but are not necessarily applicable to all samples; their relevance depends on the biobank's purpose and the intended use of the samples. In addition to the set of CDEs defined by the TEAR-RG, the biobank's data management may include other relevant information. This can encompass demographic factors such as sex, age, and ethnicity (using standard National Institutes of Health/World Health Organization categories), as well as additional variables including cosmetic use, ocular diagnoses, and systemic comorbidities.

**Table 2:** Tear fluid CDEs for collection and storage of samples.

CDEs	Values	Notes
Instructions that applied prior to tear fluid collection		

Contact lens use	No; Yes, soft; Yes, rigid	Specify wear duration
Eye drop use	No; Yes, namely …	Specify type(s) and use duration
Fluorescein use	No; Yes, namely …	Specify the time between fluorescein application and tear fluid collection
Pre-collection wash out	No; Yes, specify duration	Applies to contact lens and/or eye drop cessation

Tear fluid collection		

Collection method	Schirmer's strip, (Glass) Microcapillaries, Ocular surface wash, Swab, Sponge, Contact lens Conjunctival impression cytology, Other, namely …	Specify the brand
Collection duration	Numeric (minutes)	Measured from placement to removal
Volume collected	Volume (μL) or migration length (mm)	Report separately for OD and OS
Time of day	Morning, Afternoon, Evening, Night	Specify the exact time if possible
Date(s) of collection	DD/MM/YYYY	Multiple dates if applicable
Region of collection	Inferior/superior meniscus, Medial/lateral canthus, Other, namely …	Specify the exact region
Tear fluid type	Basal, Reflex, Emotional	
Anaesthesia use	No; Yes, namely …	Record dose and timing
Reflex induction	None, Chemical, Mechanical, Other, namely …	Only in the case of reflex tear fluid
Eye laterality	OD, OS	Required
Pooling	No, OD and OS separate; Yes	
Adverse events	No; Yes, namely …	Specify if applicable

Tear fluid storage		

Time between collection and interim storage	Time in minutes	If applicable, record elapsed time
Time in interim storage	Time in minutes	If applicable, record elapsed time
Interim storage condition	Cool block, Wet ice, Dry ice, Not applicable, Other, namely …	If applicable
Time between collection and final storage	Time in minutes	Record elapsed time
Final storage condition	−20 °C, −80 °C, −150 °C, Liquid nitrogen, Other, namely …	Required
Storage duration	Time in days, months, or years	Record elapsed time

**Abbreviations:** CDEs, common data elements; OD, oculus dexter; OS, oculus sinister.

**Table 3:** Tear fluid CDEs for processing and analysis of biobanked samples.

CDEs	Values	Notes
Pre-analytical sample processing		

Freeze-thaw cycles	Count (numeric)	If applicable
Shipping condition	Dry ice, Liquid nitrogen, Dry shipper, Other, namely …	Required in case of sample transfers
Extraction/elution buffer	PBS, TBS, Tris, Kit buffer, Other, namely …	If applicable
Extraction/elution buffer volume	Numeric (μL)	If applicable
Buffer additives	Protease/phosphatase inhibitors, RNase inhibitor, None, Other, namely …	If applicable
Sample incubation with buffer	Time (minutes)	If applicable
Centrifugation	Time (minutes) and speed (rpm)	If applicable
Concentration method	Ultrafiltration, SpeedVac, Precipitation, Other, namely …	If applicable

Tear fluid analysis		

Analyte type	Proteins, Antibodies, Nucleic acids, Drugs, Lipids, Metabolites, Extracellular vesicles, Other, namely …	Multiple analytes possible
Detection technique	ELISA, LC-MS/MS, RT-PCR, HPLC, NGS, Flow cytometry, Western blot, Other, namely …	Multiple techniques possible
Sample input volume	Numeric (μL)	Volume per well/lane etc.
Dilution factor	Numeric (ratio)	If applicable, specify dilution agent
Detection outcome	Detected, Below Limit of Detection, Not detectable, Other, namely …	Required for transparency
Storage of left over samples	No; Yes, namely.	Specify the sample number and volume

Tear fluid data analysis		

Normalization approach	Total tear fluid volume, Total protein concentration, None, Other, namely …	Required for transparency

**Abbreviations:** CDEs, common data elements; PBS, phosphate-buffered saline; TBS, Tris-buffered saline; ELISA, enzyme-linked immunosorbent assay; LC-MS/MS, liquid chromatography-tandem mass spectrometry; RT-PCR, reverse transcription-polymerase chain reaction; HPLC, high performance liquid chromatography; NGS, next-generation sequencing.

In addition to following the Tear Fluid CDEs, the use of Standard PREanalytical Code, such as "TER" for tears,^48^ is recommended to ensure consistent reporting of data in biobank files. It is also advised to align with the Findable, Accessible, Interoperable, and Reusable (FAIR) principles, as incorporated in the proposed Tear Fluid CDEs. To further improve interoperability, local variables (e.g., "eye drops Y/N") can be mapped to standardized vocabularies such as SNOMED CT,^49^ International Classification of Diseases,^50^ or Unified Medical Language System.^51^ Automated data quality checks are recommended to identify missing values and implausible entries, such as negative age values or mismatched storage conditions.

## 9 Conclusions

This review outlined the key considerations for establishing a tear fluid biobank or incorporating tear fluid samples into an existing biobank. Setting up such an infrastructure requires considerable preparation and investment, given the numerous organizational, technical, and ethical aspects involved. Once implemented in a structured and standardized manner, however, a biobank can provide long-term value by ensuring access to high-quality, well-documented samples for future research. The comprehensive overview presented here may serve as a first step towards the standardization of tear fluid biobanking.

## Tear Research Network Biobanking Taskforce

**Alejandra de-la-Torre;** Neuroscience Research Group (NEUROS), Neurovitae Center for Neuroscience, Institute of Translational Medicine (IMT), School of Medicine and Health Sciences, Universidad del Rosario, Bogotá, Colombia.

**William Rojas-Carabali;** Colombian Visual Science and Translational Eye Research Institute (CERI), Bogotá, Colombia.

**Erika Ponzini;** Department of Materials Science, University of Milano-Bicocca, Milano, Italy.COMiB Research Centre in Optics and Optometry, Milano, Italy.

**Juanita Cardona-López;** Neuroscience Research Group (NEUROS), Neurovitae Center for Neuroscience, Institute of Translational Medicine (IMT), School of Medicine and Health Sciences, Universidad del Rosario, Bogotá, Colombia.

**Imre Lengyel;** The Wellcome-Wolfson Institute for Experimental Medicine, School of Medicine, Dentistry and Biomedical Science, Queen's University Belfast, Belfast, UK.

**Sarah B. Sunshine;** Department of Ophthalmology and Visual Sciences, University of Maryland School of Medicine, Baltimore, MD, USA. University of Maryland Greenebaum Comprehensive Cancer Center, University of Maryland School of Medicine, Baltimore, MD, USA.

**Carlos Cifuentes-González;** Colombian Visual Science and Translational Eye Research Institute (CERI), Bogotá, Colombia. Ocular Immunology Department, Ophthalmology Research Center, SIGMA Clinic, Cali, Colombia.

**Françoise Brignole-Baudouin;** Sorbonne University, INSERM, CNRS, Institut de la Vision, Paris, France.Hospital National de la Vision des 15-20, INSERM-DGOS CIC 1423, IHU FOReSIGHT, Paris, France.Paris cité university, faculty of pharmacy, Paris, France.

**Swaminathan Sethu;** GROW Research Laboratory, Narayana Nethralaya Foundation, Bangalore, India.

## Study approval

Not applicable.

## Author contributions

The authors confirm contribution to the paper as follows: Conceptualization, Investigation, Methodology, Project administration, Visualization, Writing—original draft, Writing—review and editing: JS; Investigation, Writing—original draft, Writing—review and editing: SH, NS; Investigation, Writing—original draft, Writing—review and editing: NB, KK; Writing—review and editing: TRNBT; Conceptualization, Investigation, Methodology, Project administration, Supervision, Writing—original draft, Writing—review and editing: MG.

## Declaration of generative AI use

During the preparation of this work the authors used ChatGPT (OpenAI; GPT-5.5) solely for spelling and grammar checks of selected sentences. No medical writing or editorial assistance was used. All conceptual and intellectual contributions were made exclusively by the authors. The authors reviewed all content and take full responsibility for the final published manuscript.

## Funding

This research did not receive any specific grant from funding agencies in the public, commercial, or not-for-profit sectors.

## Declaration of competing interest

The authors declare that they have no known competing financial interests or personal relationships that could have appeared to influence the work reported in this paper.
